# The Global, Regional, and National Burdens of Cervical Cancer Attributable to Smoking From 1990 to 2019: Population-Based Study

**DOI:** 10.2196/40657

**Published:** 2022-12-23

**Authors:** Ruixia Yuan, Fang Ren, Yingying Xie, Kaixiang Li, Zhuang Tong

**Affiliations:** 1 Clinical Big Data Center the First Affiliated Hospital of Zhengzhou University Zhengzhou China; 2 Henan Academy of Medical Big Data Zhengzhou China; 3 Department of Gynecology the First Affiliated Hospital of Zhengzhou University Zhengzhou China; 4 Department of Scientific Research the First Affiliated Hospital of Zhengzhou University Zhengzhou China

**Keywords:** global burden of disease, cervical cancer, smoking women, time trends

## Abstract

**Background:**

Cervical cancer is the fourth most common cause of cancer death in women worldwide. Smoking is one of the risk factors for cervical cancer. Understanding the global distribution of the disease burden of cervical cancer attributable to smoking and related changes is of clear significance for the prevention and control of cervical cancer in key populations and for tobacco control. As far as we know, research on the burden of cervical cancer attributable to smoking is lacking.

**Objective:**

We estimated the disease burden and mortality of cervical cancer attributable to smoking and related trends over time at the global, regional, and national levels.

**Methods:**

Data were obtained from the Global Burden of Disease study website. Age-standardized rates were used to facilitate comparisons of mortality and disability-adjusted life years (DALYs) at different levels. The estimated annual percentage change (EAPC) was used to assess trends in the age-standardized mortality rate (ASMR) and the age-standardized DALY rate (ASDR). A Pearson correlation analysis was used to evaluate correlations between the sociodemographic index and the age-standardized rates.

**Results:**

In 2019, there were 30,136.65 (95% uncertainty interval [UI]: 14,945.09-49,639.87) cervical cancer–related deaths and 893,735.25 (95% UI 469,201.51-1,440,050.85) cervical cancer–related DALYs attributable to smoking. From 1990 to 2019, the global burden of cervical cancer attributable to smoking showed a decreasing trend around the world; the EAPCs for ASMR and ASDR were –2.11 (95% CI –2.16 to –2.06) and –2.22 (95% CI –2.26 to –2.18), respectively. In terms of age characteristics, in 2019, an upward trend was observed for age in the mortality of cervical cancer attributable to smoking. Analysis of the trend in DALYs with age revealed an initially increasing and then decreasing trend. From 1990 to 2019, the burden of disease in different age groups showed a downward trend. Among 204 countries, 180 countries showed downward trends, 10 countries showed upward trends, and the burden was stable in 14 countries. The Pearson correlation analysis revealed a significant negative correlation between sociodemographic index and the age-standardized rates of cervical cancer attributable to smoking (ρ=–0.228, *P*<.001 for ASMR and ρ=–0.223, *P*<.001 for ASDR).

**Conclusions:**

An increase over time in the absolute number of cervical cancer deaths and DALYs attributable to smoking and a decrease over time in the ASMR and ASDR for cervical cancer attributable to smoking were observed in the overall population, and differences in these variables were also observed between countries and regions. More attention should be paid to cervical cancer prevention and screening in women who smoke, especially in low- and middle-income countries.

## Introduction

Cervical cancer is the fourth most common cause of cancer death in women worldwide [1]. It is estimated that in 2020, more than 600,000 new cases of cervical cancer and as many as 342,000 cervical cancer–related deaths occurred around the world [2]. Although rare in the population younger than 15 years, cervical cancer occurs in women of all ages, with the highest incidence observed in women aged 40 to 60 years [3]. It thus poses a serious threat to women’s health. Persistent infection with human papillomavirus (HPV), especially types 16 and 18, is the main cause of cervical cancer [4,5]. Socioeconomic conditions [6], smoking, exposure to secondhand smoke, factors related to sexual behavior and childbirth, inflammation of the female reproductive system, and a family history of malignant tumors have also been identified as risk factors for cervical cancer [7-9].

Among the above risk factors, we focus here on the influence of smoking. First, smoking is a known independent risk factor for cervical cancer. A meta-analysis of the results of 9 studies with a low risk of bias [10] showed a significant correlation between smoking and cervical cancer (odds ratio [OR] 3.05, 95% CI 1.73-5.38). Second, smoking increases the rate of persistent high-risk HPV infection [11]. Third, smoking has been reported to reduce survival among patients with cervical cancer [12]. In 2019, the age-standardized prevalence of smoking among women aged 15 years or older was 6.62%, and smoking was identified as the cause of 1.51 million deaths, or 5.84% of all deaths, in this population [13].

It is particularly important to pay attention to key groups of patients with cervical cancer [14]. To the best of our knowledge, studies on the disease burden of cervical cancer attributable to smoking, especially the spatiotemporal trends, are still lacking. An understanding of the temporal and spatial changes in this burden at the global level would significantly enhance the development of active prevention and control measures tailored to local conditions. In this study, we used data from the Global Burden of Disease (GBD) study to estimate the global burden of cervical cancer attributable to smoking from 1990 to 2019 and describe changes in this burden over time. Our study aimed to answer the following questions: (1) How much of the disease burden of cervical cancer is attributable to smoking in the female population, and how is this burden changing? (2) Are the distribution and trends of the disease burden of cervical cancer attributable to smoking different in different countries and regions of the world, and are these differences related to the level of socioeconomic development? and (3) What are the age characteristics of the disease burden of cervical cancer attributable to smoking?

## Methods

### Study Data

The data used in this study were obtained from the 2019 GBD study, which provides a comprehensive and systematic assessment of the global burdens of 369 diseases, injuries, and impairments and 87 risk factors in 204 countries and territories during the 1990 to 2019 period. For this study, we extracted data on the burden of cervical cancer attributable to smoking, including mortality (calculated as the number of cervical cancer deaths × 100,000 / female population) [15] and disability-adjusted life years (DALYs; the sum of years of life lost due to premature mortality and years lived with disability) [16], at the global, regional, and national levels.

In the current study, spatial division of the sample was achieved using 3 GBD division methods. The first method is based on the sociodemographic index (SDI), a comprehensive index used to measure the level of social development in a geographic area. The included countries and territories were divided by SDI into 5 superregions: low SDI, low-middle SDI, middle SDI, high-middle SDI, and high SDI. The second method involves dividing the world into 21 geographic regions according to epidemiological similarity and geographical proximity (eg, East Asia, Australasia, and Central Europe). The third method involves simple division by country and territory (for a total of 204 countries). Furthermore, the sample population of women aged 30 to 79 years was divided into 5-year age groups (eg, 30-34 years), and those aged 80 years and older were combined into one group, to yield 11 age groups.

### Definition of Smoking Exposure

According to the GBD risk factor collaborators [17], smoking exposure is defined as the current or previous use of any smoking tobacco product other than electronic cigarettes or vaporizers. For current smokers, exposure was estimated using 2 continuous measures: the number of cigarettes per smoker per day and the number of pack-years. For former smokers, exposure was estimated using the number of years since smoking cessation.

### Statistical Analysis

In the GBD study, population data from the report *World Population Prospects, 2012 Revision* was used for standardization [18]. Age-standardized rates (ASRs) were used to eliminate the influence of different population groups and differences in the age structure of populations over time, thus ensuring the comparability of statistical indicators. We used absolute numbers and ASRs (per 100,000 people) with 95% uncertainty intervals (UIs) to describe the burden and mortality of cervical cancer attributable to smoking. We further used the age-standardized mortality rate (ASMR) and age-standardized DALY rate (ASDR) to compare the burden of cervical cancer attributable to smoking over time and between regions.

The estimated annual percentage change (EAPC) was used to assess trends in the ASMR and ASDR. The natural logarithm of the ASR was fitted to the following regression line model: ln (ASR) = α + βx + ɛ, where x is the calendar year. EAPCs and 95% CIs were derived from the following regression model: y = 100 × (exp (β) − 1), where y is the EAPC [19]. An ASR was considered to increase during the observation period when the lower limit of the 95% CI of the EAPC was greater than zero, and to decrease when the upper limit of the 95% CI was less than zero; otherwise, the ASR was considered stable. A Pearson correlation analysis was used to evaluate the correlations between SDI and ASRs.

The methods used to estimate disease burdens from indicators in the GBD have been described previously [16,20]. All statistical analyses were performed using R (version 4.1.3; R Foundation for Statistical Computing). A 2-sided *P* value <.05 was considered to indicate a statistically significant difference.

### Ethical Considerations

This study was approved by the Ethics Committee of Scientific Research and Clinical Trials of the First Affiliated Hospital of Zhengzhou University (NO. 2020-KY-167).

## Results

### Global Burden of Cervical Cancer Attributable to Smoking in 2019 and Temporal Trends From 1990 to 2019

Detailed results on the global burden of mortality and disability-adjusted life-years due to cervical cancer attributable to smoking in 1990 and 2019 and temporal trends from 1990 to 2019 are provided in Table 1, Table 2, and Table S1 in Multimedia Appendix 1. In 2019, the numbers of female deaths and DALYs attributable to cervical cancer worldwide were approximately 280,479.04 and 8,955,012.78, respectively. Of these, 30,136.65 (95% UI 14,945.09-49,639.87) cervical cancer–related deaths and 893,735.25 (95% UI 469,201.51-1,440,050.85) cervical cancer–related DALYs were attributable to smoking.

Overall, a decreasing trend was observed in the global burden of cervical cancer attributable to smoking over the studied period. The ASMR decreased from 1.28 per 100,000 (95% UI 0.65-2.06) in 1990 to 0.69 per 100,000 (95% UI 0.35-1.14) in 2019, a decrease of 85.5%, with an EAPC of –2.11 (95% CI –2.16 to –2.06) during this period. The ASDR also decreased from 39.31 per 100,000 (95% UI 21.03-62.13) in 1990 to 20.75 per 100,000 (95% UI 10.85-33.51) in 2019, with an EAPC of –2.22 (95% CI –2.26 to –2.18).

**Table 1:** The global burden of death due to cervical cancer attributable to smoking in 1990 and 2019 and temporal trends from 1990 to 2019.

		1990					2019					Estimated annual percentage change from 1990 to 2019 (95% CI)
		Deaths, n (95% UI^a^)		Age-standardized mortality rate, n × 10^-5^ (95% UI)		Deaths, n (95% UI)			Age-standardized mortality rate, n × 10^-5^ (95% UI)			
Global burden		27,421.87 (13,984.40 to 6721.02)		1.28 (0.65 to 2.06)		30,136.65 (14,945.09 to 49,639.87)			0.69 (0.35 to 1.14)		–2.11 (–2.16 to –2.06）	
**Burden by sociodemographic index**												
	Low	1830.31 (847.48 to 3193.99)	1.43 (0.67 to 2.47)		2665.07 (1196.19 to 4655.28)			0.95 (0.44 to 1.64)		–1.52 (–1.59 to –1.44）		
	Low-middle	3986.82 (2018.69 to 6721.02)	1.28 (0.64 to 2.13)		5040.14 (2422.69 to 8955.89)			0.69 (0.34 to 1.25)		–2.25 (–2.32 to –2.08）		
	Middle	6434.95 (3243.32 to 10,665.85)	1.19 (0.58 to 1.98)		7557.27 (3565.50 to 13,116.97)			0.57 (0.27 to 1.00)		–2.69 (–2.81 to –2.57）		
	High-middle	7475.99 (3799.58 to 12,052.88)	1.27 (0.65 to 2.04)		8453.26 (4173.18 to 13,819.28)			0.79 (0.40 to 1.29)		–1.52 (–1.61 to –1.43）		
	High	7669.50 (3471.33 to 12,062.72)	1.43 (0.68 to 2.22)		6390.02 (2793.35 to 10,482.46)			0.75 (0.34 to 1.19)		–2.17 (–2.28 to –2.05）		

^a^UI: uncertainty interval.

**Table 2:** The global burden of disability-adjusted life-years due to cervical cancer attributable to smoking in 1990 and 2019 and temporal trends from 1990 to 2019.

			1990				2019				Estimated annual percentage change from 1990 to 2019 (95% CI)
			DALYs^a^, n (95% UI^b^)		Age-standardized DALY rate, n × 10^-5^ (95% UI)		DALYs, n (95% UI)		Age-standardized DALY rate, n × 10^-5^ (95% UI)		
Global burden			863,494.73 (464,325.44 to 1,365,563.86)		39.31 (21.03 to 62.13)		893,735.25 (469,201.51 to 1,440,050.85)		20.75 (10.85 to 33.51)		–2.22 (–2.26 to –2.18)
**Burden by sociodemographic index**											
	Low	61,306.72 (26,248.83 to 107,671.43)		43.51 (19.69 to 76.28)		85,793.88 (34,342.57 to 153,086.77)		27.53 (11.86 to 48.17)		–1.68 (–1.76 to –1.69)	
	Low-middle	127,334.6 (64,663.76 to 208,130.21)		37.08 (18.61 to 61.43)		150,971.38 (71,542.69 to 275,063.88)		19.69 (9.37 to 35.44)		–2.32 (–2.44 to –2.19)	
	Middle	197,494.21 (103,798.33 to 328,143.85)		33.63 (17.30 to 55.60)		216,363.48 (107,107.20 to 372,743.62)		15.65 (7.73 to 26.95)		–2.75 (–2.85 to –2.66)	
	High-middle	242,456.35 (131,242.40 to 377,394.25)		41.44 (22.49 to 64.40)		260,369.48 (137,295.16 to 409,749.35)		25.66 (13.93 to 40.26)		–1.58 (–1.66 to –1.49)	
	High	234,111.80 (117,412.53 to 360,250.81)		46.78 (24.10 to 70.67)		179,289.26 (85,852.04 to 285,069.42)		23.88 (12.15 to 36.84)		–2.27 (–2.38 to –2.16)	

^a^DALY: disability-adjusted life-year.

^b^UI: uncertainty interval.

### Burden by SDI Superregion

Analysis of the 5 superregions divided by SDI revealed that the absolute number of cervical cancer deaths attributable to smoking ranged from 2665.07 (95% UI 1196.19-4655.28) in the low-SDI region to 8453.26 (95% UI 4173.18-13,819.28) in the high-middle–SDI region. The lowest and highest absolute numbers of DALYs were 85,793.88 (95% UI 34,342.57-153,086.77) in the low-SDI region and 260,369.48 (95% UI 137,295.16-409,749.35) in the high-middle–SDI region, respectively.

The highest ASMR and ASDR values were 0.95 per 100,000 (95% UI 0.44-1.64) and 27.53 per 100,000 (95% UI 11.86-48.17), respectively, in the low-SDI region, and the lowest ASMR and ASDR values were 0.6 per 100,000 (95% UI 0.35-1.14) and 15.65 per 100,000 (95% UI 7.73-26.95), respectively, in the middle-SDI region.

Downward trends in the ASMR and ASDR were observed in all 5 SDI superregions from 1990 to 2019. The largest declines were observed in the middle-SDI region, with EAPCs of –2.69 (95% CI –2.81 to –2.57) and –2.75 (95% CI –2.85 to –2.66) for the ASMR and ASDR, respectively. The smallest declines in the ASMR and ASDR were observed in the low-SDI and high-middle–SDI regions, respectively, with EAPCs of –1.51 (95% CI –1.59 to –1.44) and –1.58 (95% CI –1.66 to –1.49).

### Burden by Geographic Region

Among the 21 geographic regions, the largest absolute numbers of cervical cancer–related deaths and DALYs attributable to smoking in 2019 were observed in East Asia, and the smallest absolute numbers were observed in Oceania. In the same year, the highest ASMR was 2.84 per 100,000 (95% CI 1.33-5.01), and the highest ASDR was 95.03 per 100,000 (95% CI 52.82-142.33), observed in Oceania and southern Latin America, respectively, whereas the lowest ASMR was 0.23 per 100,000 (95% CI 0.09-0.43), and the lowest ASDR was 6.71 per 100,000 (95% CI 3.05-11.69), observed in North Africa and the Middle East, respectively.

Downward trends in the cervical cancer burden attributable to smoking were observed in most geographic regions from 1990 to 2019. The largest decrease was observed in Central Latin America, with an EAPC of –4.52 (95% CI –4.73 to –4.30) for the ASMR, and in tropical Latin America, with an EAPC of –4.38 (95% CI –4.58 to –4.17) for the ASDR. In contrast, an upward trend was observed in Eastern Europe, with EAPCs of 0.76 (95% CI 0.45-1.07) and 1.2 (95% CI 0.85-1.55) for the ASMR and ASDR, respectively.

### Burden by Country

**Figure 1:**
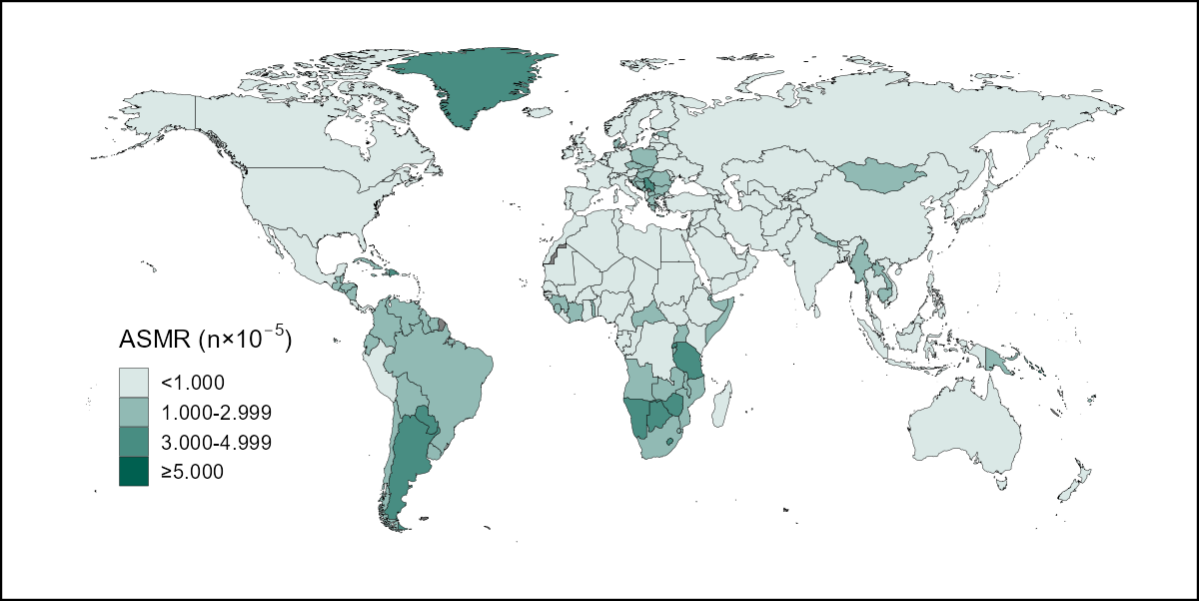
The global distribution of ASMRs of cervical cancer attributable to smoking in 2019. ASMR: age-standardized mortality rate.

**Figure 2:**
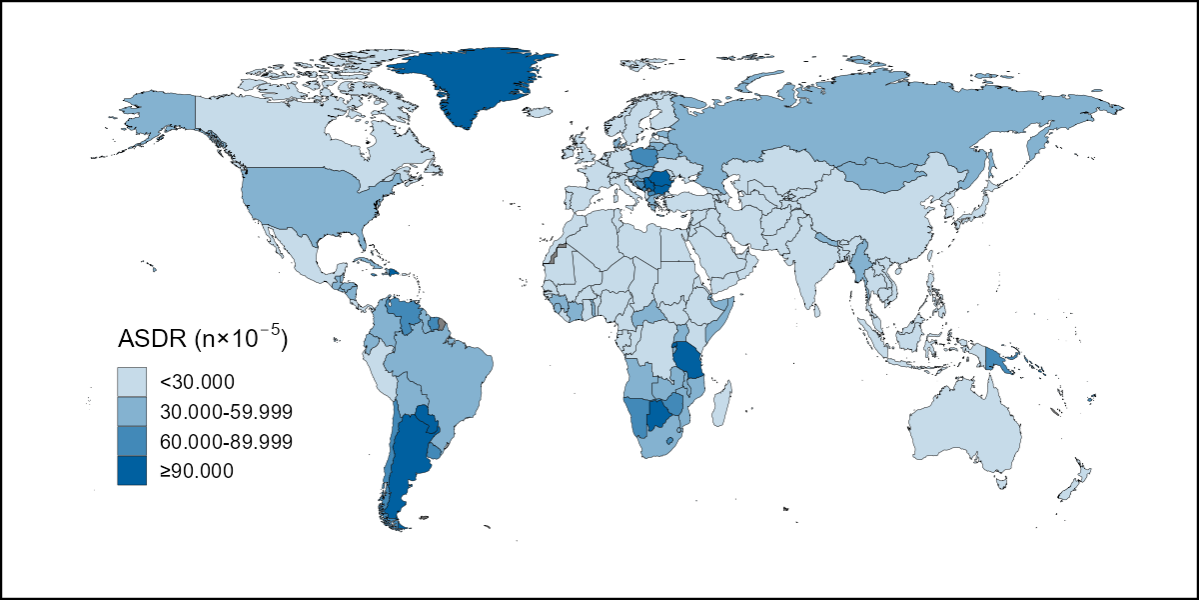
The global distribution of ASDRs of cervical cancer attributable to smoking in 2019. ASDR: age-standardized disability-adjusted life-year rate.

In 2019, the highest ASMR of cervical cancer attributable to smoking across all 204 included countries and territories was 483.2 times the lowest ASMR; the highest value of 24.16 per 100,000 (95% CI 13.27-38.83) was observed in Kiribati, and the lowest value of 0.05 per 100,000 (95% CI 0.02-0.11) was observed in Egypt. Kiribati and Egypt also had the highest and lowest ASDRs, at 734.33 per 100,000 (95% CI 385.97-1,184.35) and 1.17 per 100,000 (95% CI 0.39-2.57), respectively. Figures 1 and 2 show maps depicting the burden and mortality of cervical cancer attributable to smoking in 2019 in the 204 included countries, with darker and lighter colors indicating more or less severe conditions, respectively. Briefly, high burdens of cervical cancer attributable to smoking were observed in countries in South Asia, southern Africa, southern South America, and Greenland in North America, whereas low burdens were observed in countries in Europe, North America, and Oceania.

**Figure 3:**
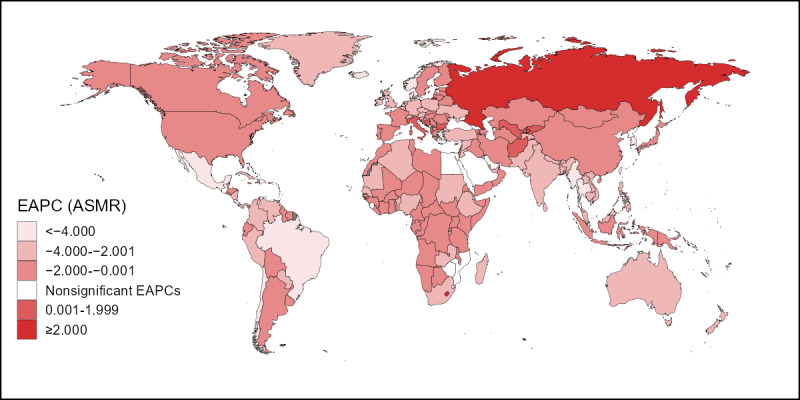
EAPCs in the ASMR of cervical cancer attributable to smoking by country from 1990 to 2019. EAPC: estimated annual percentage change; ASMR: age-standardized mortality rate.

**Figure 4:**
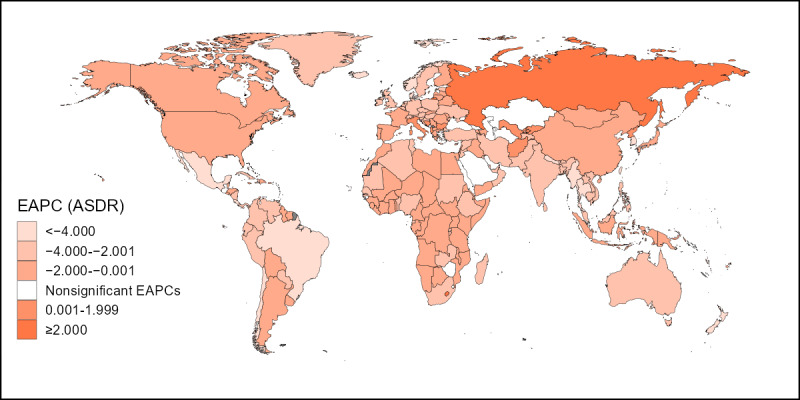
EAPCs in the ASDR of cervical cancer attributable to smoking by country from 1990 to 2019. EAPC: estimated annual percentage change; ASDR: age-standardized disability-adjusted life-year rate.

A downward trend in the ASMR of cervical cancer attributable to smoking was observed in most countries over time (ie, the EAPC was less than zero and the upper limit of the 95% CI was less than zero), with the strongest trends observed in Mexico, Thailand, Singapore, the Maldives, and Denmark. In contrast, upward trends in the ASMR (ie, the EAPC was greater than zero and the lower limit of the 95% CI was greater than zero) were observed in 10 countries over time (listed in descending order according to the EAPC value): Lesotho, the Russian Federation, Bulgaria, Afghanistan, Albania, Uzbekistan, Italy, Kyrgyzstan, Bosnia and Herzegovina, and Guinea-Bissau. In addition, 16 countries showed stable ASMR over time (the 95% CI included zero). Similarly, downward trends in the ASDR were observed in most countries over time. However, upward trends were observed in Lesotho, the Russian Federation, Bulgaria, Afghanistan, Albania, Uzbekistan, Italy, Kyrgyzstan, Bosnia and Herzegovina, and the Democratic People’s Republic of Korea, while ASDR was stable in 14 countries. Figures 3 and 4 depict the degree of change in the burden of cervical cancer attributable to smoking from 1990 to 2019. In the figures, darker colors indicate a stronger downward trend and uncolored areas on the map indicate stability.

### Characteristics of and Temporal Changes in the Cervical Cancer Burden Attributable to Smoking by Age

In 2019, an upward trend was observed in the mortality of cervical cancer attributable to smoking with age. The highest mortality rate was observed in the population older than 80 years, except for a low value in the group aged 75 to 79 years (Multimedia Appendix 2, Figure S1). Analysis of the trend in DALYs with age revealed an initially increasing and then decreasing trend, with the highest DALYs observed in the group aged 55 to 59 years (Multimedia Appendix 3, Figure S2).

Figures S3 and S4 in Multimedia Appendices 4 and 5 present the EAPCs of the ASRs of cervical cancer attributable to smoking for all age groups over the 1990 to 2019 period. Decreasing trends were observed in the ASRs of mortality and DALYs. The most obviously decreasing trend was observed in the group aged 35 to 39 years, with EAPCs of –3.19 (95% CI –3.36 to –3.03) and –3.18 (95% CI –3.35 to –3.01) for ASMR and ASDR, respectively.

### Correlations Between SDI and EAPCs of the ASRs

Figures S5 and S6 in Multimedia Appendices 6 and 7 present scatterplots of correlations between SDI and EAPCs of the ASDR and ASMR in 2019. A Pearson correlation analysis revealed a significant negative correlation between SDI and the ASMR and ASDR of cervical cancer attributable to smoking (ρ=–0.228, *P*<.001 for ASMR and ρ=–0.223, *P*<.001 for ASDR).

## Discussion

### Principal Findings

Our study estimated time trends in the burden of cervical cancer attributable to smoking from 1990 to 2019 at the global, regional, and national levels. In terms of spatial distribution, the worldwide distribution of this burden in 2019 was quite different in each country and region. In terms of time trends, 10 countries showed increasing trends from 1990 to 2019 and 14 countries were stable. These countries were distributed in Europe, Central Asia, and South Africa; other countries and regions showed decreasing trends.

The burden of cervical cancer related to smoking in the whole population showed an increase in the absolute number of cases and a decrease over time for the ASMR and ASDR, as well as differences in different countries and regions. This may be related, first, to the distribution characteristics of female smokers and female smoking rates around the world. Global smoking prevalence among women aged 15 years or older declined by 37.7% in 2019 compared with 1990, but population growth led to an increase in the total number of female smokers. There were 146 million current female smokers aged 30 years or older in 2019. At the same time, the prevalence of smoking among women aged 15 years or older varied greatly in different countries and regions, with the age-standardized prevalence of smoking tobacco use ranging from 0.696% in Eritrea to 42.3% in Greenland [13]. Differences in the prevalence of smoking among women around the world inevitably led to differences in the burden of disease attributable to smoking, including cervical cancer.

Second, the implementation of tobacco control measures may also have affected the spatial distribution characteristics and temporal trends of the burden of cervical cancer attributable to smoking. In 2005, the World Health Organization (WHO) Framework Convention on Tobacco Control (FCTC) entered into force [21], and based on a consensus on the importance of tobacco control, many countries and regions around the world ratified the treaty [22]. In 2017, Gravely et al [23] used WHO data from 126 countries to examine the association between the highest levels of implementation of key demand-reduction measures of the WHO FCTC from 2007 to 2014 and smoking prevalence from 2005 to 2015. Gravely concluded that the decline in smoking prevalence was closely related to the implementation of key tobacco-control measures over the treaty’s first decade and suggested that tobacco-related morbidity and mortality will continue to decline in the future. However, it cannot be ignored that there are huge gaps in the implementation of tobacco control in different countries and regions [24]. Moreover, there is a gender blind spot in tobacco control action, and the problems and challenges of female smoking are not fully recognized, especially in low- and middle-income countries [25].

Tobacco control is one of the most important cancer prevention behaviors. However, for smokers, a focus on cancer screening has the greatest potential benefit because it allows the timely detection of disease and early intervention [26]. Although smokers are at higher risk for some types of cancer, Byrne et al [27] showed that smokers are less accepting of cancer-related screening than nonsmokers. Therefore, in order to continue to reduce the burden of cervical cancer attributable to smoking, it is necessary, first, to continue to implement tobacco control measures while paying full attention to gender, so as to reduce women’s tobacco use and improve women’s status, and second, to further improve cervical cancer screening methods, so as to increase the screening rate for female smokers.

According to our study, the distribution of and trends in the burden of cervical cancer attributable to smoking also differed in different age groups. Mortality due to cervical cancer related to smoking increases with age, which may be related to longer exposure to smoking [28]. DALYs due to cervical cancer attributable to smoking were highest in the group aged 55 to 64 years, which had the largest absolute number of patients among age groups. Compared with patients in older age groups, the decreasing trend in the burden of cervical cancer attributable to smoking has been more pronounced in younger age groups over the last 3 decades. This may be a benefit of the promotion of cervical cancer vaccines, the continuous improvement of screening measures, and the implementation of tobacco control measures.

The results of this study show that the level of socioeconomic development is correlated with the burden of cervical cancer attributable to smoking and trends in this burden. First, countries or regions with different economic development levels have different levels of burden of cervical cancer attributable to smoking. In 2019, this burden was mainly concentrated in low- and middle-SDI countries in southern Africa, South America, and Asia. This is consistent with the global distribution of the overall burden of cervical cancer; 86% of the global burden of cervical cancer is in Africa, Latin America and the Caribbean, and Asia [29]. Therefore, it is necessary to pay special attention to the fact that although the female smoking rate is relatively low in low- and middle-income countries and regions, the burden of cervical cancer cannot be ignored. Secondly, SDI has a negative correlation with trends in this burden in the past 3 decades, which may be related to the more obvious effect of some protective factors in countries or regions with higher economic development levels. These protective factors include more effective tobacco control [30], better screening methods for cervical cancer in women, more widespread screening coverage, and more secure medical resources [31].

### Study Limitations

This study has some limitations. First, the availability of certain data on the disease burden of cervical cancer attributable to smoking in low-income countries is poor, while the GBD study relied heavily on existing epidemiological studies to estimate global disease prevalence. However, all national data calculation standards were consistent, so data quality was guaranteed. Second, the GBD study did not present data on the disease burden of cervical cancer from second-hand smoke (ie, household or occupational exposure), which is an important mode of tobacco exposure in women. If this risk factor were taken into account, the disease burden of cervical cancer due to tobacco would be higher than the current estimate, and the trend might also be different. Third, we only estimated the burden of cervical cancer attributable to smoking, but the combined effects of smoking and other risk factors may increase or complicate the burden of cervical cancer.

### Conclusion

The distribution of the disease burden of cervical cancer attributable to smoking and trends in this burden differ in different countries and regions of the world. More attention should be paid to cervical cancer prevention and screening in women who smoke, especially in low- and middle-income countries.

## Appendix

Multimedia Appendix 1 The global burden and mortality of cervical cancer attributable to smoking in 1990 and 2019 and temporal trends from 1990 to 2019 (Geographic Regions).

Multimedia Appendix 2 Age-specific rates of global cervical cancer deaths attributable to smoking in 2019.

Multimedia Appendix 3 Age-specific rates of global cervical cancer DALYs attributable to smoking in 2019. DALY: disability-adjusted life-year.

Multimedia Appendix 4 EAPCs in ASMR of cervical cancer attributable to smoking by age group from 1990 to 2019. EAPC: estimated annual percentage change; ASMR: age-standardized mortality rate.

Multimedia Appendix 5 EAPCs in ASDR of cervical cancer attributable to smoking by age group from 1990 to 2019. EAPC: estimated annual percentage change; ASDR: age-standardized disability-adjusted life-year rate.

Multimedia Appendix 6 The correlations between the SDI index and EAPCs of the ASMR. ASMR: age-standardized mortality rate; EAPC: estimated annual percentage change.

Multimedia Appendix 7 The correlations between the SDI index and EAPCs of the ASDR. ASDR: age-standardized disability-adjusted life-year rate; EAPC: estimated annual percentage change.

## Abbreviations

ASDR: age-standardized disability-adjusted life-year rate
ASMR: age-standardized mortality rate
ASR: age-standardized rate
DALY: disability-adjusted life-year
EAPC: estimated annual percentage change
FCTC: Framework Convention on Tobacco Control
GBD: Global Burden of Disease
SDI: sociodemographic index
UI: uncertainty interval
WHO: World Health Organization

## Data Availability

The datasets generated and analyzed during this study are available in the Global Burden of Disease (GBD) study 2019 data repository (https://ghdx.healthdata.org/gbd-results-tool).
