# Supplementary material for: The Global, Regional, and National Burdens of Cervical Cancer Attributable to Smoking From 1990 to 2019: Population-Based Study
Source: JMIR Public Health Surveill. 2022 Dec 23;8(12):e40657. doi: 10.2196/40657 (PMC9823574; doi:10.2196/40657)
Supplement: Multimedia Appendix 1 [file publichealth_v8i12e40657_app1.docx]

Table S1a The global burden and mortality of cervical cancer attributable to smoking in 1990 and 2019 and temporal trends from 1990 to 2019 (Deaths) ( Geographic Region)

| Characteristics | 1990 | | 2019 | | 1990-2019 |
| --- | --- | --- | --- | --- | --- |
|  | NO.(95%UI) | ASMR NO.×10^-5^(95%UI) | NO.(95%UI) | ASMR  NO.×10^-5^(95%UI) | EAPC(95%CI） |
| Regions |  |  |  |  |  |
| East Asia | 3257.50（1504.48-6180.00） | 0.73(0.33-1.38) | 5551.63（2213.48-10339.73） | 0.50(0.20-0.94) | -1.00（-1.23，-0.77） |
| Southeast Asia | 1983.89（966.74-3399.43） | 1.42(0.69-2.49) | 2025.49（920.08-3862.93） | 0.61(0.28-,1.18) | -3.23（-3.43，-3.03） |
| Oceania | 64.31（29.89-106.29） | 3.73(1.79-6.33) | 118.38（52.96-206.50） | 2.84(1.33-5.01) | -0.93（-0.98，-0.88） |
| Central Asia | 162.42（77.26-288.68） | 0.58(0.28-1.04) | 193.81（91.04-332.79） | 0.41(0.19-0.70) | -0.69（-0.98，-0.40） |
| Central Europe | 2397.63（1233.00-3732.27） | 3.09(1.62-4.73) | 1977.79（954.73-3190.27） | 2.07(1.08-3.24) | -1.37（-1.49，-1.25） |
| Eastern Europe | 1127.67（537.71-1933.69） | 0.71(0.35-1.22) | 1345.85（682.22-2297.74） | 0.83(0.42-1.40) | 0.76（0.45,1.07） |
| High-income Asia Pacific | 909.58（358.47-1603.86） | 0.82(0.33-1.44) | 811.69（312.90-1492.89） | 0.43(0.18-0.73) | -2.16（-2.19，-2.12） |
| Australasia | 151.08（73.04-234.56） | 1.27(0.62-1.93) | 122.06（54.25-204.85） | 0.53(0.25-0.86) | -2.67（-3.08，-2.26） |
| Western Europe | 4254.96（1949.83-6700.09） | 1.49(0.74-2.29) | 3106.02（1424.20-5053.07） | 0.78(0.39-1.22) | -2.00（-2.13，-1.88） |
| Southern Latin America | 991.29（562.01-1478.55） | 4.00(2.29-5.95) | 1175.34（638.13-1788.21） | 2.79(1.55-4.22) | -1.41（-1.57，-1.25） |
| High-income North America | 2525.03（1107.61-3920.24） | 1.45(0.66-2.22) | 2763.77（1122.97-4540.02） | 0.95(0.41-1.54) | -1.45（-1.58，-1.31） |
| Caribbean | 410.16（207.19-647.85）） | 2.99(1.49-4.76) | 539.89（259.64-896.20） | 1.98(0.96-3.29) | -1.48（-1.59，-1.37） |
| Andean Latin America | 167.29（70.35-301.03） | 1.47(0.62-2.67) | 247.46（99.76-471.69） | 0.83(0.34-1.58) | -2.02（-2.13，-1.92） |
| Central Latin America | 1573.22（814.58-2468.19） | 3.43(1.83-5.41) | 1385.49（663.57-2419.39） | 1.06(0.51-1.86) | -4.52（-4.73，-4.30） |
| Tropical Latin America | 2756.04（1449.19-4153.04） | 5.31(2.70-8.08) | 2277.38（1009.92-3874.26） | 1.69(0.75-2.89) | -4.24（-4.44，-4.04） |
| North Africa and Middle East | 360.56（160.71-642.74） | 0.39(0.17-0.71) | 528.93（232.64-944.88） | 0.23(0.09-0.43) | -1.80（-1.91，-1.69） |
| South Asia | 2445.52（1144.83-4226.97） | 0.83(0.41-1.45) | 3141.37（1500.16-6064.89） | 0.43(0.20-0.84) | -2.29（-2.39，-2.19） |
| Central Sub-Saharan Africa | 147.17（57.62-302.27） | 1.03(0.41-2.12) | 263.06（103.63-526.61） | 0.78(0.31-1.56) | -1.03（-1.07，-0.99） |
| Eastern Sub-Saharan Africa | 764.59（270.36-1432.52） | 1.88(0.73-3.51) | 1277.58（449.75-2417.23） | 1.40(0.55-2.59) | -1.13（-1.19，-1.06） |
| Southern Sub-Saharan Africa | 617.29（301.73-1050.49） | 3.81(1.82-6.52) | 731.48（353.01-,1251.67） | 2.22(1.04-3.78) | -1.77（-2.08，-1.46） |
| Western Sub-Saharan Africa | 354.66（158.30-670.84） | 0.80(0.36-1.50) | 552.17（225.39-1039.14） | 0.51(0.21-0.96) | -1.56（-1.65，-1.47） |

Table S1b The global burden and mortality of cervical cancer attributable to smoking in 1990 and 2019 and temporal trends from 1990 to 2019 (DALYs)

( Geographic Region)

| Characteristics | 1990 | | 2019 | | 1990-2019 |
| --- | --- | --- | --- | --- | --- |
|  | NO.(95%UI) | ASDR  NO.×10^-5^(95%UI) | NO.(95%UI) | ASDR  NO.×10^-5^(95%UI) | EAPC(95%CI） |
| Region |  |  |  |  |  |
| East Asia | 87601.60（40390.76-167380.53） | 18.48（8.49-35.31） | 151037.61（61756.33-281199.53） | 13.57（5.64-25.24） | -0.67（-0.92，-0.43） |
| Southeast Asia | 60178.76（29186.29-102778.37） | 39.44（19.26-67.13） | 56727.67（24511.57-106980.33） | 16.06（7.05-29.99） | -3.44（-3.64，-3.24） |
| Oceania | 2315.14（1013.12-3871.84） | 122.36（56.86-201.76） | 4293.23（1808.90-7576.56） | 92.13（40.95-160.39） | -0.96（-1.02，-0.90） |
| Central Asia | 5550.12（2607.61-9909.95） | 19.97（9.42-35.83） | 7092.09（3307.56-12479.18） | 14.44（6.76-25.31） | -0.55（-0.86，-0.24） |
| Central Europe | 82056.43（44922.83-123145.59） | 109.93（61.50-164.17） | 61733.28（33212.99-96377.69） | 70.85（39.16-108.34） | -1.56（-1.69，-1.42） |
| Eastern Europe | 38002.34（18846.42-65209.89） | 25.61（12.01-43.71） | 49995.63（25605.52-84686.01） | 33.05（16.55-55.33） | 1.20（0.85,1.55） |
| High-income Asia Pacific | 27484.01（12116.01-47531.17） | 25.06（11.12-42.91） | 21365.19（9137.69-36686.06） | 13.84（6.43-23.43） | -1.90（-1.99,-1.85） |
| Australasia | 4809.78（2568.47-7165.51） | 42.20（22.88-62.33） | 3517.66（1760.74-5607.84） | 17.18（8.45-27.45） | -2.81（-3.25，-2.37） |
| Western Europe | 124315.59（62678.80-188664.11） | 48.75（25.95-72.24） | 85411.00（43621.28-130713.27） | 25.09（13.36-37.67） | -2.08（-2.19，-1.96） |
| Southern Latin America | 34723.64（19779.88-50503.74） | 142.21（80.96-205.79） | 38205.22（21113.56-57154.68） | 95.03（52.82-142.33） | -1.61（-1.76，-1.45） |
| High-income North America | 82439.74（40672.37-123271.40） | 50.34（25.07-74.79） | 81155.76（36629.95-129114.97） | 31.21（14.86-48.85） | -1.69（-1.84，-1.55） |
| Caribbean | 13497.48（7134.42-21392.01） | 95.82（50.41-151.46） | 16027.48（7894.38-26602.13） | 59.84（29.38-99.66） | -1.78（-1.93，-1.64） |
| Andean Latin America | 5627.57（2211.53-9934.29） | 46.55（18.67-82.58） | 7572.76（2776.05-14380.56） | 24.79（9.11-47.31） | -2.24（-2.36，-2.13） |
| Central Latin America | 50360.68（24710.28-79960.05） | 100.09（50.17-158.79） | 43048.52（19092.76-76316.02） | 32.41（14.52-,57.06） | -4.33（-4.55，-4.10） |
| Tropical Latin America | 91639.00（51575.70-136571.10） | 163.24（89.93-242.40） | 67647.08（31835.24-113452.08） | 50.18（23.59-84.14） | -4.38（-4.58，-4.17） |
| North Africa and Middle East | 11971.39（5542.78-20868.10） | 11.99（5.47-20.97） | 16733.03（8019.22-28709.94） | 6.71（3.05-11.69） | -2.07（-2.17，-1.97） |
| South Asia | 79266.10（36623.95-136519.68） | 24.42（11.56-41.94） | 92737.30（42718.70-174529.01） | 12.07（5.58-22.74） | -2.49（-2.61，-2.38） |
| Central Sub-Saharan Africa | 5171.04（2035.17-10535.42） | 33.42（13.16-68.03） | 9151.58（3462.33-18626.97） | 24.66（9.57-49.70） | -1.09（-1.14，-1.05） |
| Eastern Sub-Saharan Africa | 25038.84（7733.35-46955.18） | 55.32（18.48-103.75） | 41041.89（13138.96-77717.63） | 39.95（13.57-75.04） | -1.26（-1.33，-1.19） |
| Southern Sub-Saharan Africa | 20263.06（9994.00-33928.71） | 117.21（58.70-199.29）） | 21115.15（10184.24-35777.21） | 60.49（29.33-103.33） | -2.13（-2.39，-1.86） |
| Western Sub-Saharan Africa | 11182.41（4709.14-21301.95） | 23.32（10.01-44.51） | 18126.11（7371.37-34284.50） | 14.68（6.01-27.49） | -1.61（-1.72，-1.50） |
