# Supplementary figures and images for: The Global, Regional, and National Burdens of Cervical Cancer Attributable to Smoking From 1990 to 2019: Population-Based Study
Source: JMIR Public Health Surveill. 2022 Dec 23;8(12):e40657. doi: 10.2196/40657 (PMC9823574; doi:10.2196/40657)

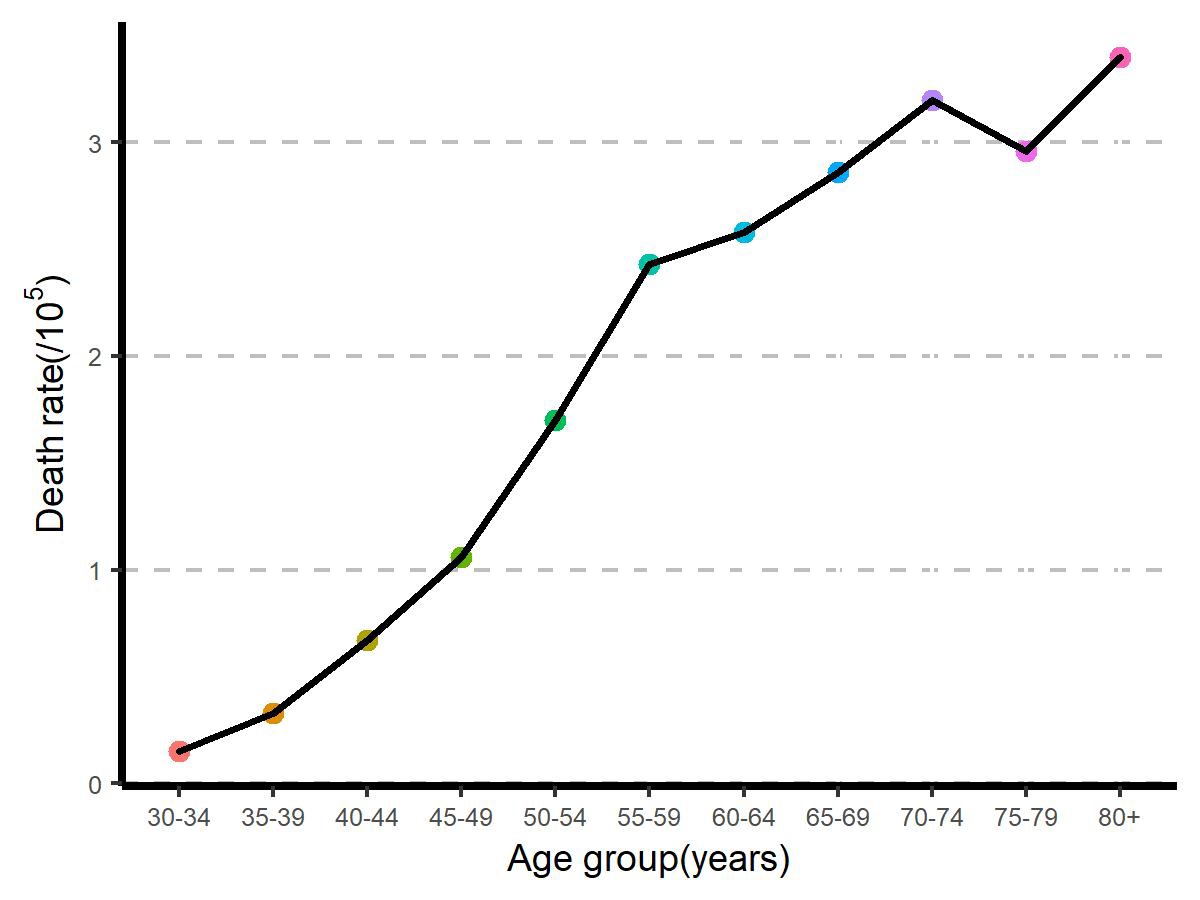

Supplement: Multimedia Appendix 2 [file publichealth_v8i12e40657_app2.png]

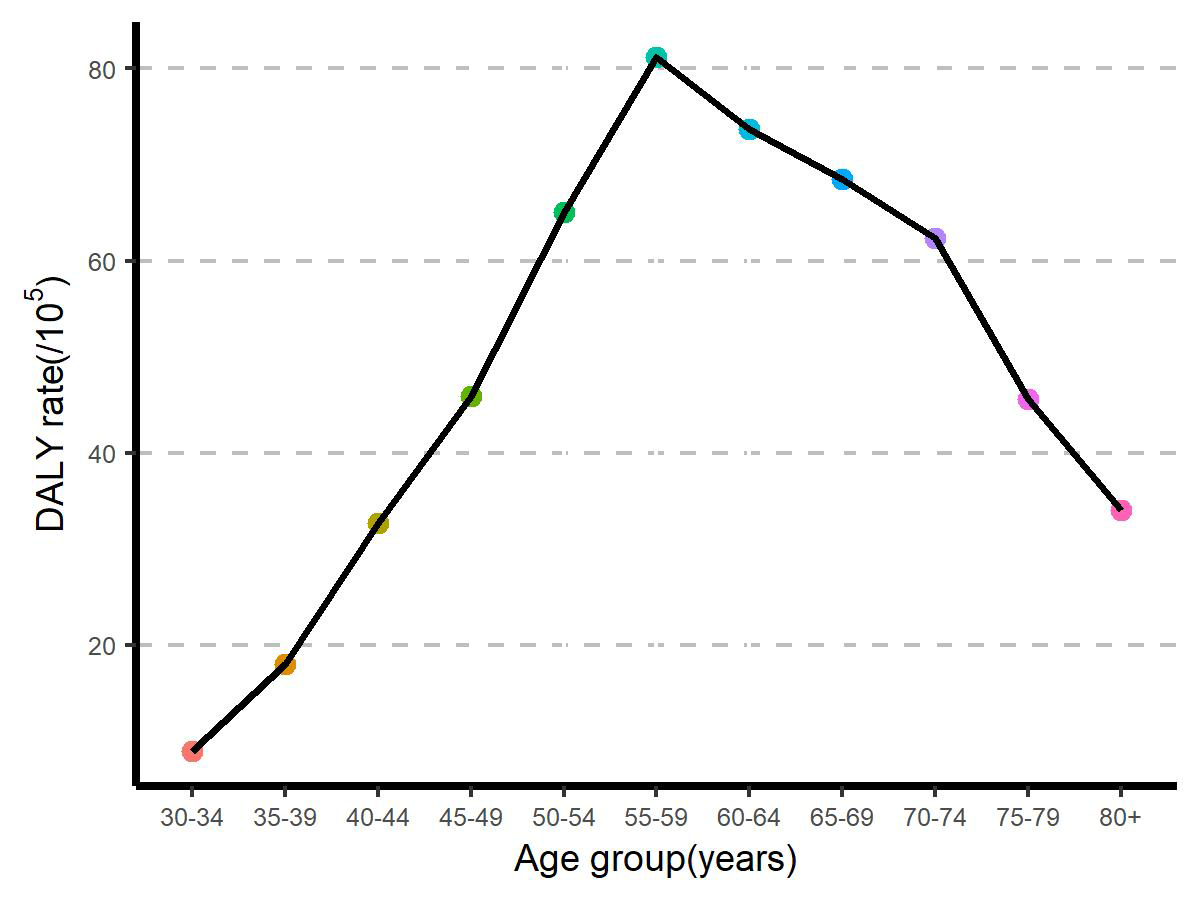

Supplement: Multimedia Appendix 3 [file publichealth_v8i12e40657_app3.png]

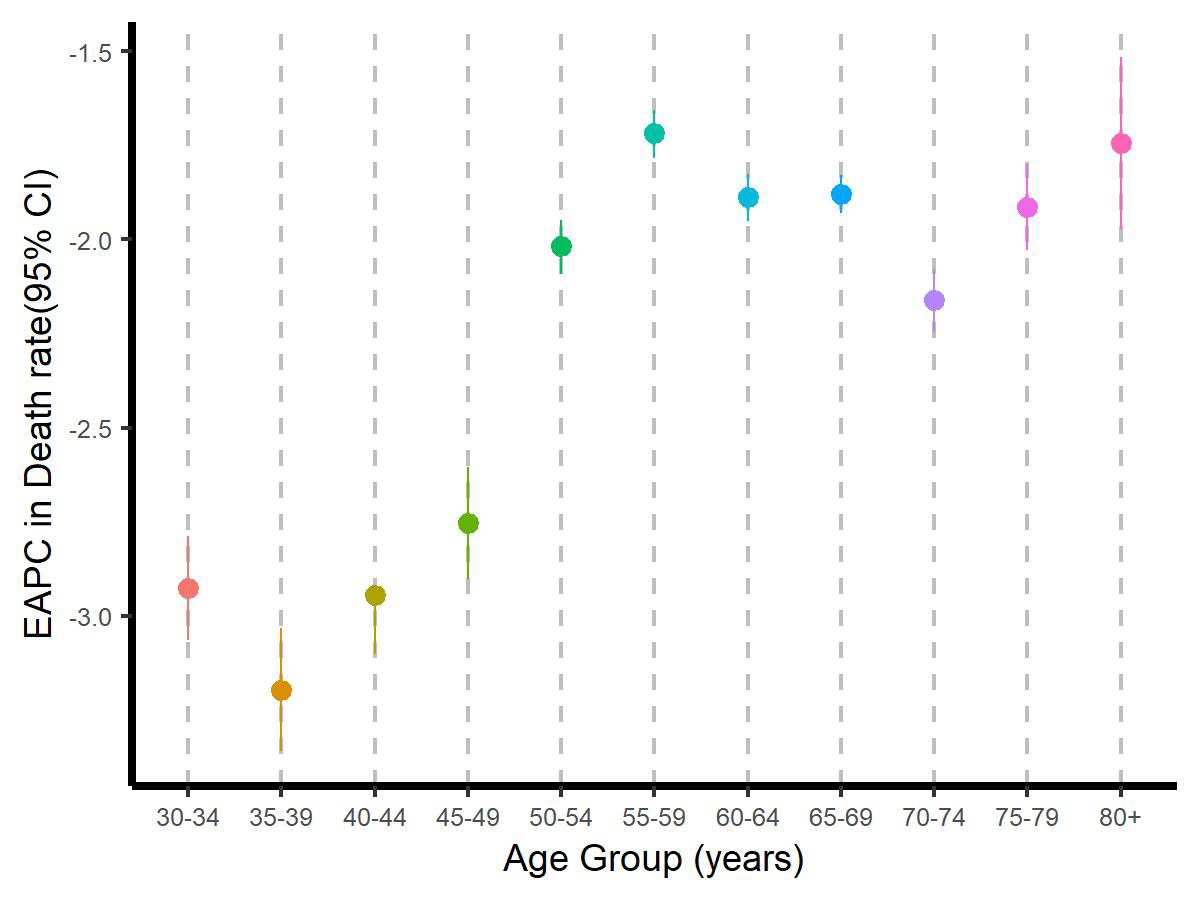

Supplement: Multimedia Appendix 4 [file publichealth_v8i12e40657_app4.png]

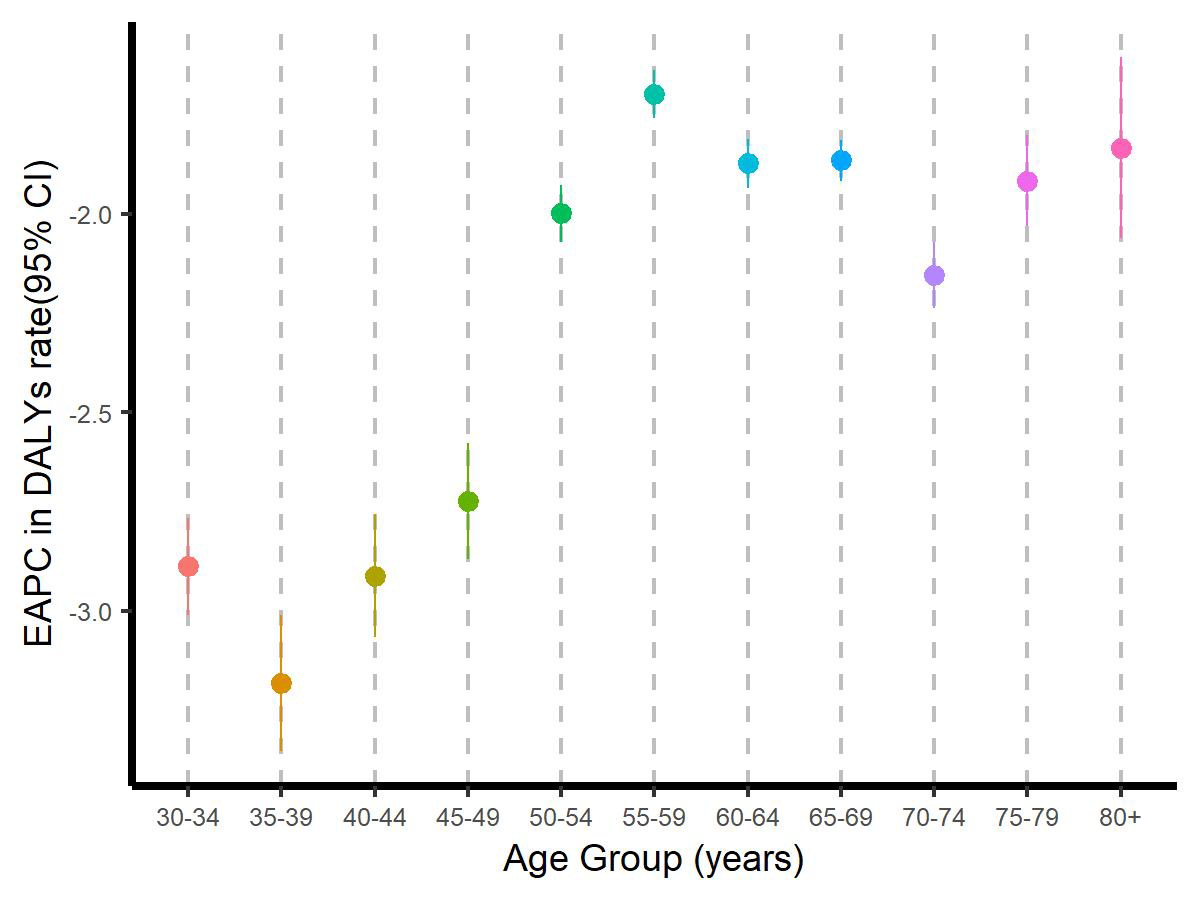

Supplement: Multimedia Appendix 5 [file publichealth_v8i12e40657_app5.png]

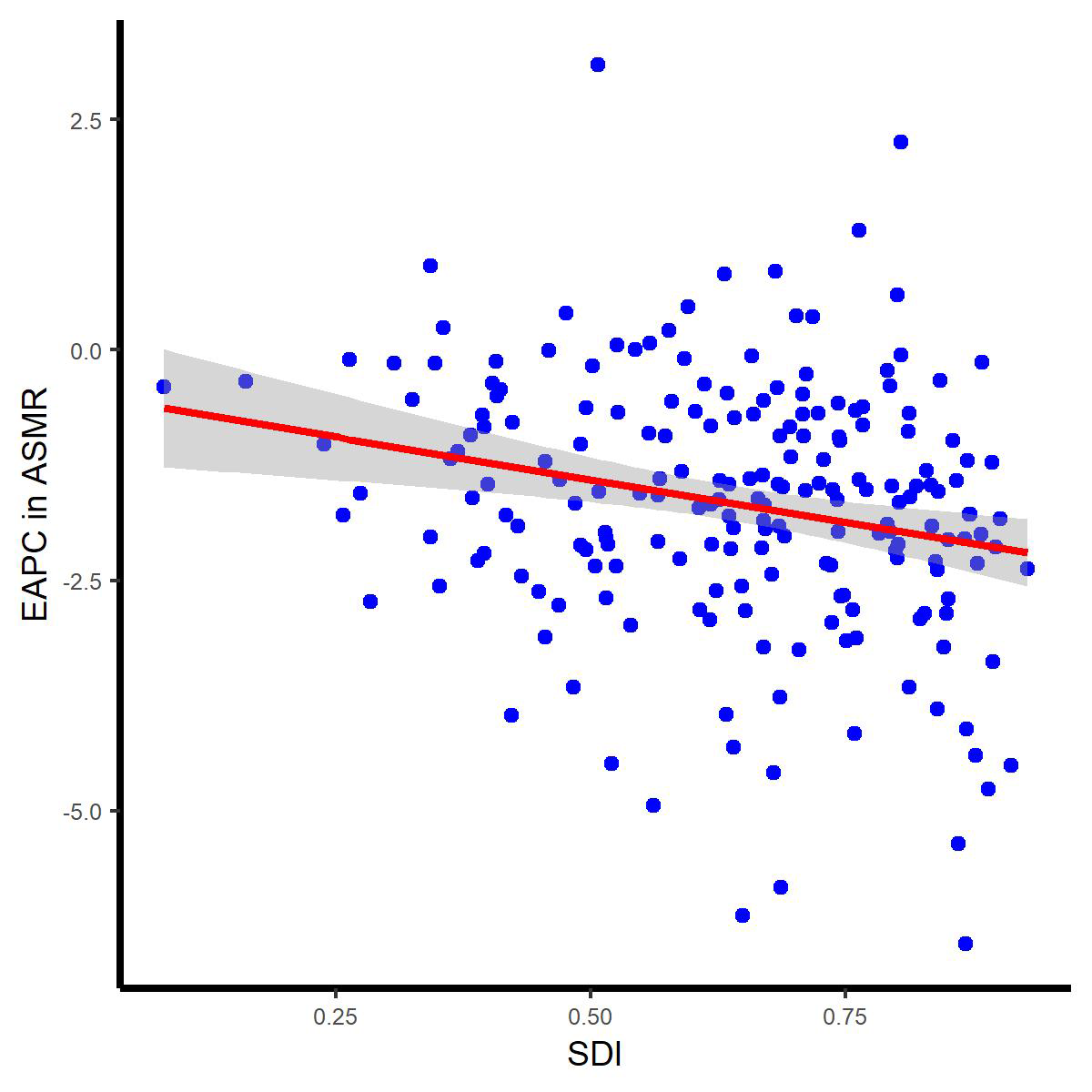

Supplement: Multimedia Appendix 6 [file publichealth_v8i12e40657_app6.png]

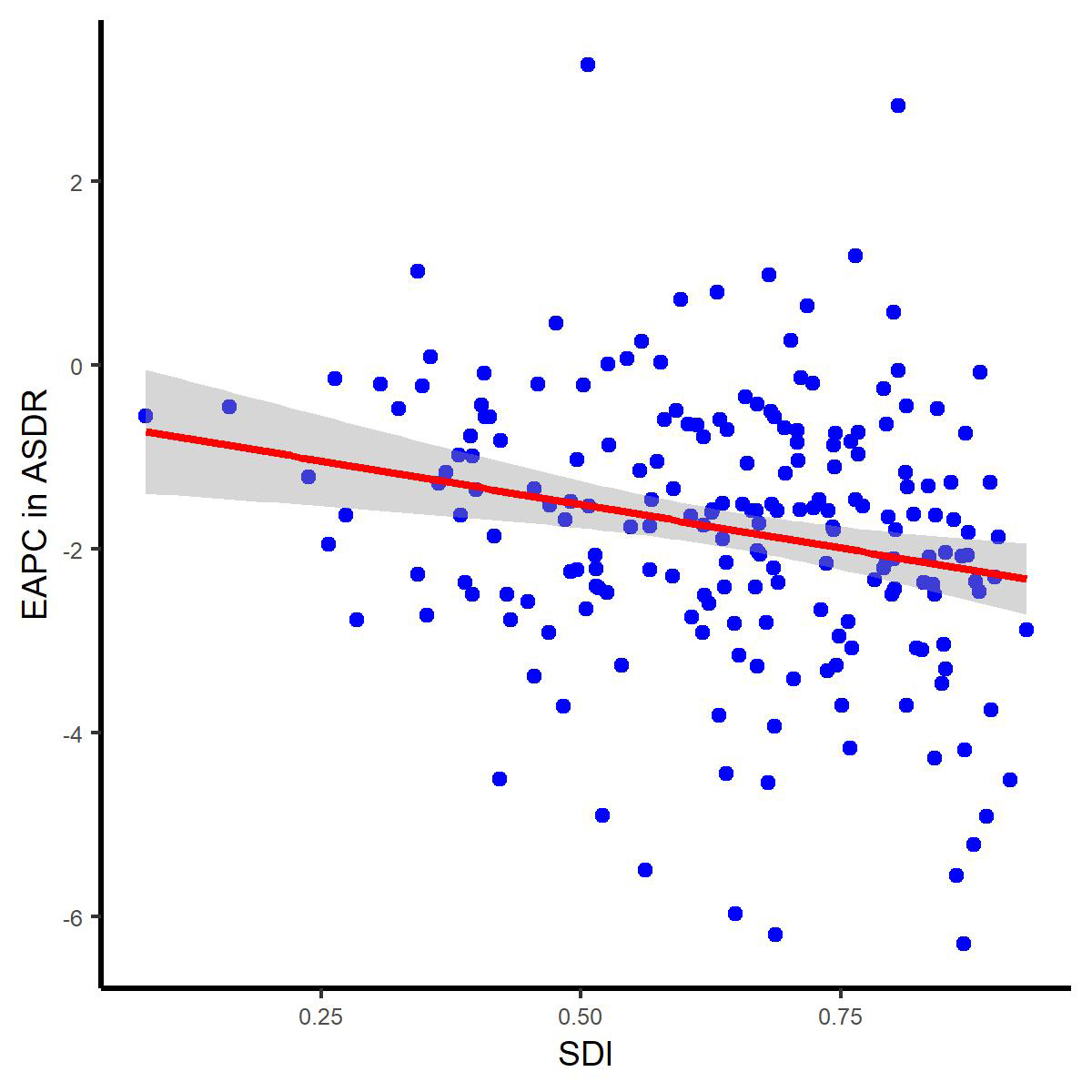

Supplement: Multimedia Appendix 7 [file publichealth_v8i12e40657_app7.png]
